# Multiscale design, interaction mechanism and performance of CL-20/Al energetic composites with embedded structure†

**DOI:** 10.1039/d0ra07602c

**Published:** 2020-12-18

**Authors:** Jun Tao, Xiaofeng Wang, Hao Wang, Kun Zhang

**Affiliations:** Xi'an Modern Chemistry Research Institute Xi'an 710065 Shaanxi People's Republic of China taojun4712230@126.com wangxf_204@163.com

## Abstract

In order to design and prepare hexanitrohexaazaisowurtzitane/aluminum (CL-20/Al) composites, the contact state of CL-20 with aluminum particles under different mixed solvent contents were calculated with the method of dissipative particle dynamics (DPD). Then the modified attachment energy (AE) model was applied to predict the morphologies of CL-20 in ethyl acetate, hexane and ethyl acetate/hexane mixed solvents. Furthermore, the morphologies, the surface element distribution, the sensitivity and the energy performance of CL-20/Al composites prepared with the solvent/non-solvent method were characterized, and the interaction mechanism were also obtained. The results achieved show that phase separation phenomenon becomes obvious with the decrease of ethyl acetate/*n*-hexane mixed solvent content. CL-20/aluminum particles will form the composites of aluminum particles embedded in CL-20 crystal when the solvent content is zero. The order of modified attachment energies for ethyl acetate/hexane mixed solvents on CL-20 faces is (011) > (110) > (101) ≈ (11−1) ≈ (10−1) > (002). Besides that, the crystalline morphology of CL-20 in acetate/hexane mixed solvents is spindle-shaped. There are many Al particles embedded in CL-20 crystals of CL-20/Al composites prepared by using solvent/non-solvent method. The calculated results agree well with the experimental results. In CL-20/Al composites, aluminum particles interact with CL-20 mainly through hydrogen bond and strong van der Waals force. The sensitivity of CL-20/Al composites decreased obviously compared with pure CL-20 and mechanical mixing composites. Besides, the CL-20/Al composites with embedded structure can increase the explosion reaction temperature to 791.2 K, which has obvious energy advantage compared with CL-20/Al physical mixture.

## Abstract 2

In order to design and prepare hexanitrohexaazaisowurtzitane/aluminum (CL-20/Al) composites, the contact state of CL-20 with aluminum particles under different mixed solvent contents were calculated with the method of dissipative particle dynamics (DPD).

## Graphical abstract

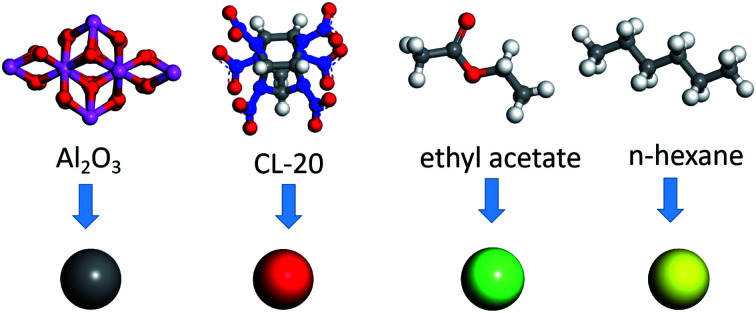

## 1 Introduction

With the rapid progress in science and technology, new war equipment and space equipment are constantly emerging, and new requirements for the performance of various kinds of equipment are put forward in the military and space fields.^1,2^ Therefore, the performance of energetic materials must be continuously improved. Composite energetic materials hierarchical and novel assembly structures composed of energetic materials, which are encapsulated, embedded and combined with another or more energetic materials through chemical bonds or intermolecular forces. Combustion and explosion properties of energetic materials are influenced by their micro-size structure. Composite energetic materials, owing to their short-distance contact between components, can greatly promote the heat and mass transfer process of materials, increase the energy release rate of the system, and reduce the sensitivity of materials. Therefore, the preparation of composite energetic materials has become a hot issue in recent years.^3–7^

Among composite energetic materials, Al-based composite energetic materials are composed of Al particles and metal, non-metal oxides, organic compounds, *etc.* The contact between reactant particles in Al-based composite energetic materials is kept closer, which reduces the distance of mass and heat transfer in the reaction process.^8–10^ It has the advantages of low ignition temperature and energy, fast energy release rate, high energy utilization rate, fast combustion rate and high pressure output.^11,12^ In recent years, researchers have begun to prepare energetic composites of explosive/aluminum powder, to realize the close contact between aluminum powder and explosives, to improve the concentration of explosive/aluminum powder per unit volume, and to improve the reaction rate and reaction completeness of aluminum powder. Yang *et al.*^13^ prepared a core–shell energetic composites of KClO_4_@Al/CuO by coating Al/CuO nano-composite particles with KClO_4_ by using solvent/non-solvent method. The Al/CuO composites are uniformly mixed by mechanical ball milling process, and CuO was used as metal oxide catalyst. The flame sensitivity of the composites is much higher than that of traditional similar materials, and the burning rate is three times higher than that of traditional nano Al/CuO. Zhigach *et al.*^14^ prepared HMX/Al nanocomposites with 50 nm aluminum powder by using suspension atomization and mechanical dry mixing method. It was found that the nano-energetic composites prepared by suspension atomization were loose solid powders, and the HMX crystals would grow during the suspension atomization process. When the pressure range was 3−10 Mpa, the burning rate of the pressed samples increased from 19 mm s^−1^ to 55 mm s^−1^, and the pressure exponent was in the range of 0.34–0.84. In addition, the nano-energetic composites of RDX, HMX and CL-20 and Al were aslo prepared by using spray drying method.^15^ The nano aluminum was dispersed into the explosive solution to form suspension liquid. Then the nano-energetic composites with explosive particles of 1 μm were obtained. Slocik *et al.*^16^ prepared Al/ammonium perchlorate (AP) nano-energetic composites through layer by layer self-assembly method. AP can interact with the surface of nano Al particles after modified by ferritin, which shortens the mass transfer and diffusion distance in the reaction. The burning rate of Al/AP energetic composites is the fastest when the number of assembly layers is 1, and the combustion rate is 160 ms. Therefore, through the previous work, it can be seen that energetic composites can increase the reaction completeness and reaction rate of aluminum powder by reducing the diffusion distance and increasing the contact area between components. However, there is only a simple physical contact between CL-20 and Al particles in the CL-20/Al energetic composite reported in the present literature. Compared with the common physical mixture, the particle size of aluminum powder is only reduced from micron scale to nano scale. Moreover, the use of nano size aluminum powder will improve the sensitivity of energetic composite on the one hand, and the activity of aluminum powder will be limited on the other hand. Therefore, it is necessary to innovate the structure and preparation method of CL-20/Al energetic composites, further increasing the contact area between CL-20 and aluminum powder particles, and obtaining energetic composite with excellent energy and safety.

In this paper, the mesoscopic morphologies of CL-20 with aluminum particles were calculated by dissipative particle dynamics (DPD) method, and the attachment energy (AE) model was applied to predict the crystal morphologies of CL-20 in solvents. Then CL-20/Al composites were prepared by using solvent/non-solvent method. The morphology, surface element distribution, sensitivity, energy performance and interaction mechanism of CL-20/Al composites were researched. CL-20/Al composites with embedded structure has obvious advantages of energy and sensitivity, whose application prospect is very good. The results are helpful to provide certain reference for the preparation and design of CL-20/Al composites.

## 2 Experimental and computational methods

### 2.1 Selection of force field

COMPASS force field was selected to simulate CL-20 based energetic composites.^17,18^ The reasons are as follows: firstly, most parameters of COMPASS force field are determined by *ab initio* data. The *ab initio* method is used to obtain the intramolecular bond parameters. Simultaneously, the empirical method based on condensed molecular dynamics is used to optimize the non-bonding parameters. COMPASS force field is a relatively perfect molecular force field at present. Secondly, the optimized configuration of RDX using COMPASS force field is in good agreement with the quantum mechanics results, and the predicted RDX cell structure based on COMPASS force field is also in good agreement with the experiment.^16–18^ The potential function of COMPASS force field is a three-term potential energy function. Compared with the traditional force filed, it can describe the potential energy surface more precisely. Herein, the stretching energy is shown in Formula (1):

1 *E*_b_ = *k*_2_(*b* − *b*_0_)^2^ + *k*_3_(*b* − *b*_0_)^3^ + *k*_4_(*b* − *b*_0_)^4^

The bending energy and twist energy of the bond angle also adopt three force field constants of the sum of three terms. Several cross terms are added to the stress field, including bond angle bending/torsion, bond stretching/torsion, bond stretching/bond stretching and so on.^17,18^ As for the non-bonding energy term, the van der Waals energy of the force field adopts the Lennard-Jones-9–6 function, and the specific expression is shown in Formula (2):2
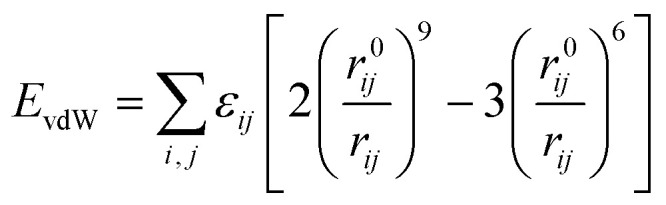


The MD simulated results by the COMPASS force field are listed in Table 1. It is found that our calculated results for the lattice parameters agree well with the experiments. Their deviations are 1.29%, 2.48% and 1.56% for *a*, *b*, and *c*, and 4.73% for *ρ* of ε-CL-20, respectively. Besides, the deviations are 0.04%, 0.04% and 0.22% for *a*, *b*, and *c*, and 0.12% for *ρ* of Al_2_O_3_, respectively. The small discrepancy suggests that the COMPASS force field is appropriate to simulate the chosen crystal systems here.

**Table 1:** Applicability of COMPASS force field to CL-20 and Al_2_O_3_

System	Data analysis	*a*	*b*	*c*	*α*	*β*	*γ*	*ρ*
CL-20	COMPASS	8.727	12.250	13.149	90	105.337	90	2.147
	Experimental^19^	8.841	12.562	13.358	90	106.820	90	2.050
	Deviation (%)	1.29	2.48	1.56	0	1.39	0	4.73
Al_2_O_3_	COMPASS	4.761	4.761	12.962	90	90	120	3.992
	Experimental^20,21^	4.759	4.759	12.991	90	90	120	3.987
	Deviation (%)	0.04	0.04	0.22	0	0	0	0.12

### 2.2 Parameters and calculation of dissipative particle dynamics (DPD) model

**Fig. 1:**
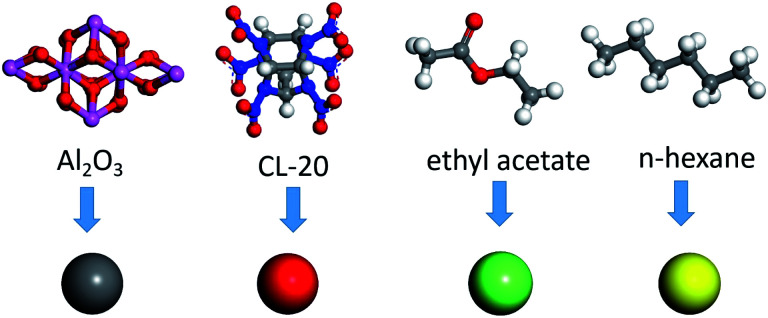
Coarse-grained models of CL-20, Al_2_O_3_, acetate and hexane.

There is a layer of dense Al_2_O_3_ film on the surface of aluminum powder. To understand the miscibility of CL-20 and aluminum powder in solvents is essential to study the miscibility of CL-20 and Al_2_O_3_. Herein, dissipative particle dynamics (DPD) was used to study the miscibility of the components. In the DPD model, the coarse-grained model was used for CL-20, Al_2_O_3_, acetate and hexane. As displayed in Fig. 1, a CL-20 molecule was coarsened into red beads, expressed by bead C; an Al_2_O_3_ cluster was coarsened into gray beads, expressed by bead O; an ethyl acetate molecule was coarsened into green beads, expressed by bead Y; a *n*-hexane molecule was coarsened into green beads, expressed by bead Z.

The key to establishing DPD is to obtain the interaction parameters between the four kinds of beads. The repulsion force parameter *a*_*ij*_ is obtained based on the Flory Huggins mixing theory. The density of the simulation system is set as *ρ* = 3, and the interaction parameter between similar beads is *a*_*ii*_ = 25*k*_b_*t*. The interaction parameter *a*_*ij*_ between different beads can be calculated by formula a_*ii*_ ≈ a_*ii*_ + 3.27*χ*_*ij*_. The calculation results are shown in Table S1.†

### 2.3 Crystal morphology predicted by AE model

Based on the periodic bond-chain (PBC) model, the AE model for crystal growth was established. The PBC model assumes that (i) the time required for bonding to the crystalline surface is inversely proportional to the bonding energy, (ii) the growth rate of the crystal is proportional to the bonding energy, (iii) the crystal is composed of PBC, (iv) the direction of the strongest bonds is according to the fastest crystal growth direction. In the AE model, the crystal morphology can be obtained by analyzing the distance between the center and the plane of the dominant crystal surface, and the distance between the center and the plane of the distribution is related to the relative growth rate. The attachment energy (*E*_att_) is the energy released by adding a growth slice to a growing crystalline surface.^17,18^

In the model, considering the influence of the solvent layer, the calibration factor *E*_s_ for vacuum attachment energy is introduced, which describes the binding energy of solvent on the (*hkl*) face of HMX crystal. It can be calculated as follows:3
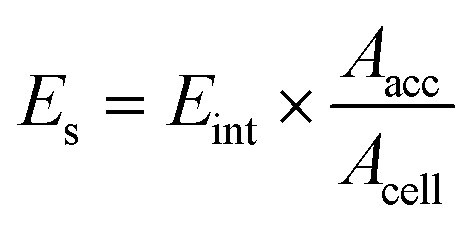
here, *A*_acc_ is the solvent-accessible area of the crystal face in the unit cell.^14,19^*A*_cell_ is the total crystal face area along the (*hkl*) plane.

The solvent attachment energy can be described using Formula (4):4
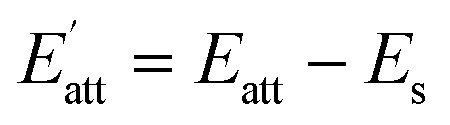


The growth rate of each crystal face 
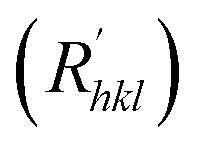
 is proportional to the solvent attachment 
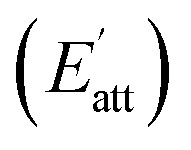
 energy of the crystal (as shown in Formula (5)), which is raised by Hartman in the modified morphology theoretic.5
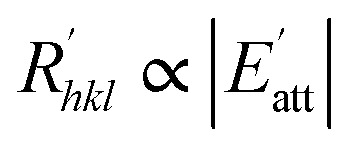


### 2.4 Molecular dynamics simulation

The ε-CL-20 original cell was built,^19^ optimizing the original structure using the forceit module. Then, the AE model was applied to predict the growth morphology in vacuum, and the main growth faces (*hkl*) were obtained. A 3 × 3 × 3 super cell was built based on the optimized original cell, cutting crystal along (011), (10−1), (110), (11−1), (002) and (101) face, respectively. A 30 Å vacuum layer was added to each face of ε-CL-20. Then the Atom Volumes & Surfaces tool of the MS software was applied to calculate the solvent-accessible area, in which there was a field whose value at each point in space corresponded to the depth in the nearest Connolly probe of a given radius, as it rolled over the van der Waals surface of the atomistic structure.

The amorphous cell module was applied to build the solvent layer containing 200 random acetate molecules with a target density of 0.900 g cm^−3^, the solvent layer containing 200 random hexane molecules with a target density of 0.692 g cm^−3^, and the solvent layer containing 50 random acetate molecules and 150 random hexane molecules with a target density of 0.734 g cm^−3^. The size of the periodic cell for the acetone layer must consistent with the size of the CL-20 periodic cell. Further geometry optimization and MD simulation in the *NPT* ensemble were carried out for acetone molecules by using the Andersen thermostat.

The adsorption models for CL-20 face-acetate, CL-20 face-hexane and CL-20 face-acetate/hexane were built based on a CL-20 layer and a solvent (acetate, hexane and acetate/hexane) layer. The solvent layer was placed along the *c* axis on the CL-20 surface and a 30 Å vacuum was built above the solvent layer. The initial configuration of CL-20-solvent was optimized, and annealing (300−500 K, 1 000 000 steps) was operated to the CL-20-solvent configuration so as to eliminate unreasonable conformations. The configurations were taken after annealing to the MD simulation in the constant-pressure and constant-temperature (*NPT*) ensemble at 298 K for 1000 ps. Also, Andersen thermostat was used.^19–22^

Then the Al_2_O_3_ original cell was built, optimizing the original structure on the basis of the forceit module. A 3 × 3 × 3 super cell was built based on the optimized original cell, cutting crystal along (001), (010) and (100) face, respectively. Three CL-20 supercells were constructed according to the surface area of Al_2_O_3_(001), Al_2_O_3_(010) and Al_2_O_3_(100) supercells, which must guarantee that CL-20 molecule could completely cover the crystal face of Al_2_O_3_. The adsorption models for Al_2_O_3_(001)-CL-20, Al_2_O_3_(010)-CL-20 and Al_2_O_3_(100)-CL-20 were built from an Al_2_O_3_ layer and a CL-20 layer. The solvent layer was placed along the *c* axis on the Al_2_O_3_ surface and a 30 Å vacuum was built above the solvent layer. The initial configuration of Al_2_O_3_-CL-20 (displayed in Fig. S1†) was optimized, and annealing (300–500 K, 1 000 000 steps) was operated to the Al_2_O_3_(100)-CL-20 configuration so as to eliminate unreasonable conformations. The configurations were taken after annealing to the MD simulation in the constant-pressure and constant-temperature (*NPT*) ensemble at 298 K for 1000 ps. Also, Andersen thermostat was used.

### 2.5 CL-20/Al composites preparation

#### 2.5.1 Materials

Al powder is commercially available. The average particle sizes are from 4 μm to 5 μm, and the purity is greater than 98%. Each batch of aluminum powder can only be used after the purity and particle size are inspected to be qualified, and the purity of aluminum powder was inspected by Chinese GB3169.1-1982. Field emission scanning electron microscope SU8010 is used to characterize the morphology and the surface elements distribution.

#### 2.5.2 Preparation of CL-20/Al composites *via* solvent/non-solvent method

80 g CL-20 was added into ethyl acetate until the CL-20 completely dissolved, adding it to the drip funnel for later use. 15 g Al was added into hexane to form Al–hexane suspension. The volume ratio of ethyl acetate and hexane was controlled at 1 : 3. The suspension was stirred, and the stirring speed was controlled at 200 rpm. Adding CL-20-ethyl acetate solution to the Al–hexane suspension and dropping out within 1 hour. The obtained suspension was filtered and dried to obtain CL-20/Al composites.

#### 2.5.3 Sensitivity test

Chinese GJB772A-97 method 601.1 was used to test the impact sensitivity.^21^ Friction sensitivity was tested according to Chinese GJB772A-97 method 602.1.^21^

#### 2.5.4 Explosion energy performance test

**Fig. 2:**
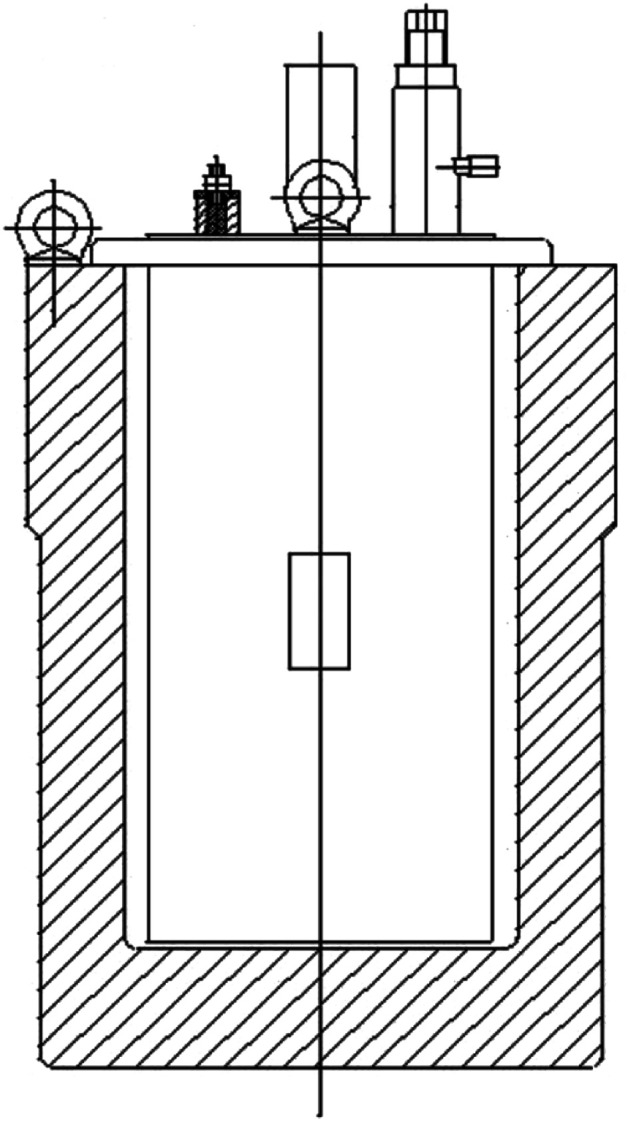
Schematic diagram of the closed explosion device.

CL-20/Al composites with embedded structure and CL-20/Al physical mixture were prepared. Then kneading granulation method was applied to prepare the aluminized explosive by adding 3% EVA and 2% paraffin. The molding powder was pressed to form grains with a diameter of 20 mm and a mass of 15 g. The blast performance was tested in a closed explosion device as shown in Fig. 2. The temperature change of explosive reaction in closed explosive device was measured.

## 3 Results and discussion

### 3.1 Contact state of CL-20 with aluminum particles with different solvent contents

There is a dense Al_2_O_3_ film on the surface of aluminum particles, and the contact between CL-20 and aluminum particles is essentially the contact with dense Al_2_O_3_ film on the surface of aluminum particles. In order to study the contact state between CL-20 and aluminum particles in mixed solvents (ethyl acetate/*n*-hexane) of different content, the mesoscopic morphologies of CL-20/Al_2_O_3_ composites were calculated with the dissipative particle dynamics (DPD) method. The mesoscopic morphology of CL-20 and aluminum particles with 80% solvent volume fraction (20% ethyl acetate and 60% *n*-hexane) are displayed in Fig. S2.† Under the condition of 80% solvent content, CL-20 can contact with Al particles in the solvent. The mesoscopic morphology changes little with time, and there is no obvious phenomenon of phase separation during the simulation. At 20 000 steps, DPD simulation can reach equilibrium state.

**Fig. 3:**
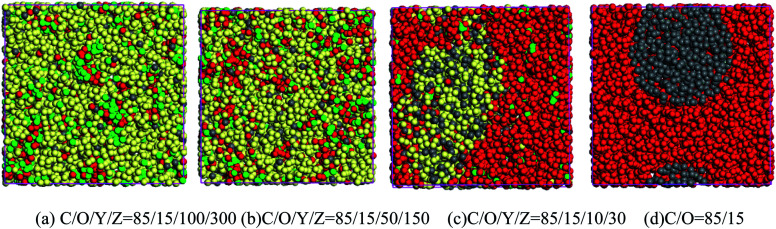
Mesoscopic morphology of CL-20/Al_2_O_3_ with different solvent fraction.

The contact state between CL-20 and aluminum powder particles under different solvent conditions was further studied, as shown in Fig. 3. With the decrease of ethyl acetate/*n*-hexane mixed solvent content, the phase separation in the system becomes gradually obvious. When the solvent content is 28%, there are two phases in the system, one is the mixture of CL-20 and ethyl acetate, and the other is a mixture of aluminum particles and *n*-hexane. When the solvent content is further reduced to 0, there are only CL-20 and Al_2_O_3_ in the system, and there are two phases in the system. The Al_2_O_3_ phase tends to form spherical aggregates embedded in the CL-20 phase.

**Fig. 4:**
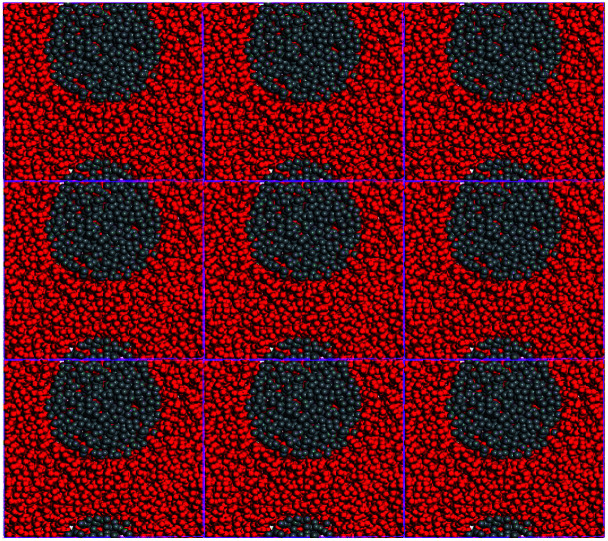
Mesoscopic morphology of CL-20 and aluminum particles with no solvent.

Furthermore, nine periodic chambers of mesoscopic morphology of CL-20/Al_2_O_3_ composites with no solvent were superimposed to form a larger mesoscopic morphology, as shown in Fig. 4. It can be seen that with the solvent filtration or evaporation, CL-20 and aluminum particles will form the composites of aluminum particles embedded in CL-20 crystal.

### 3.2 Growth morphology of hexanitrohexaazaisowurtzitane (CL-20) in solvents

**Fig. 5:**
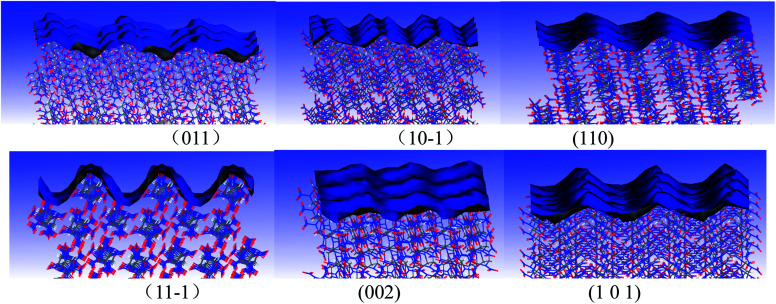
The molecular arrangement of different CL-20 crystal faces repesented by Connolly surfaces.

In order to research the crystalline morphologies of CL-20 in mixed solvents, the crystalline surface characteristics of CL-20 was studied. Faces including (011), (10−1), (110), (11−1), (002) and (101) are the main growth surfaces of CL-20. The molecular arrangements of CL-20 crystalline surfaces are displayed in Fig. 5. Herein, parameter “*S*” is introduced to describe the crystalline surface characteristics, which refers to the ratio of the solvent accessible area and the corresponding surface area. It can be seen from Table S2† that there are differences among the “*S*” values of the 6 faces of CL-20. The “*S*” value of (11−1) face is 1.48 (the biggest one), which suggests that (11−1) face is the roughest. (11−1) face can promote the adsorption of ethyl acetate and hexane molecules.

In order to understand the adsorption characteristics of ethyl acetate, hexane and ethyl acetate/hexane mixed solvents on different cystalline surfaces of CL-20, the binding energies between CL-20 and different solvents were calculated, the equilibrium conformations are shown in Fig. S3.† The predicted results are displayed in Table S3.† Take (011) face as an example. The binding energies of ethyl acetate, hexane and ethyl acetate/hexane on CL-20 (011) face are 1023.93 kJ mol^−1^, 735.64 kJ mol^−1^ and 1364.31 kJ mol^−1^, respectively, all of which are positive. It suggests that all ethyl acetate, hexane and ethyl acetate/hexane mixed solvents can adsorb on CL-20 (011) face steadily. The adsorption characteristics of ethyl acetate, hexane and ethyl acetate/hexane mixed solvents on other crystalline surfaces of CL-20 are similar to those of CL-20 (011) face.

Based on the results of the crystalline surface characteristics of CL-20 and the interaction between CL-20 and solvents, the crystalline growth parameters for CL-20 in ethyl acetate, hexane and ethyl acetate/hexane mixed solvents were calculated, as displayed in Table S4.† For ethyl acetate, the order of the solvent attachment energy for CL-20 faces is: (101) > (10−1) > (011) > (110) > (11−1) > (002). For hexane, (101) face has the greatest solvent attachment energy (*E*_s_) value (−281.41 kJ mol^−1^); (110) face has the lowest *E*_s_ value, which is only −792.19 kJ mol^−1^. There is little difference among the *E*_s_ value for hexane on different faces of CL-20. For acetate/hexane mixed solvents, the order of the *E*_s_ value for CL-20 faces is as follows: (011) > (110) > (101) ≈ (11−1) ≈ (10−1) > (002). The results suggest that the interaction intensities of the (101) face with ethyl acetate and hexane are the greatest. The solvent adsorption capability of (101) face for acetate and hexane is the strongest. The adsorption capability is mainly determined by two factors, one is the number of the exposed polar groups on the crystalline surface; and the other is the roughness of the crystal surface. However, for ethyl acetate/hexane mixed solvent, the interaction intensity of it on the (011) face is the greatest. It shows that the use of mixed solvents in the crystallization process of CL-20 is not simply a physical addition. The mixing of solvents can create different properties for CL-20 such as crystalline morphology that the single solvent can not possess.

According to the AE model theory, CL-20 interacts with solvent layers, which will change the vacuum attachment energy. Therefore, the modified attachment energy was calculated. As displayed in Table S4,† the order of modified attachment energy for ethyl acetate on CL-20 faces is: (101) > (10−1) > (011) > (110) > (11−1) > (002). Therefore, the (002) face has the greatest morphological importance for its slowest growth rate. Due to the fast growth rate, the area of (101) and (10−1) faces will reduce in the final crystalline morphology of CL-20. Similarly, the (110) face has the greatest morphological importance for CL-20 in hexane; the (002) face has the greatest morphological importance for CL-20 in acetate/hexane mixed solvents. The morphologies of CL-20 in ethyl acetate, hexane and ethyl acetate/hexane mixed solvents are displayed in Fig. S4,† which are predicted by the AE model. The shape and the area distributions of the crystalline morphology are very different for ethyl acetate, hexane and ethyl acetate/hexane mixed solvents. The crystalline morphology of CL-20 in hexane is spindle-shaped, which is very close to that in vacuum. The crystalline morphology of CL-20 in acetate/hexane mixed solvents is also spindle-shaped. However, the shape is more flat, and the proportion of the surface area of (002) face is larger.

Based on the results of miscibility calculation and morphology prediction, the morphology of CL-20/Al energetic composites was predicted. As shown in Fig. S5,† the existence of aluminum particles has little effect on the crystal morphology of CL-20. However, CL-20 will adsorb and crystallize on the aluminum powder particles. Moreover, during the growth of CL-20 crystal, aluminum particles will contact with CL-20 crystal surface under the action of mechanical energy. The aluminum particles will be embedded in the crystal. Finally, a large amount of aluminum powder embedded in the inner and surface of CL-20 crystal will be formed.

### 3.3 Preparation of CL-20/Al composites in solvents

**Fig. 6:**
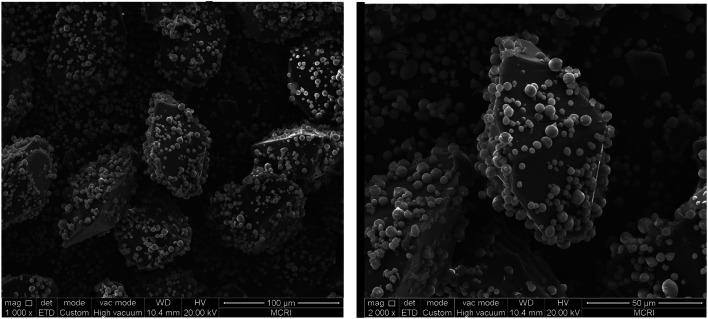
The morphology of CL-20/Al composites crystallize in acetate/hexane mixed solvent.

Solvent/non-solvent method was applied to prepare CL-20/Al composites, with acetate used as solvent, and hexane used as non-solvent, and the volume ratio of acetate/hexane was 1 : 3. The morphology of CL-20/Al composites crystallize in acetate/hexane mixed solvents is displayed in Fig. 6. It can be seen from Fig. 6 that the morphology of CL-20 in CL-20/Al composites is spindle-shaped. There are many aluminum particles embedded in CL-20 crystals, and part of aluminum particles are tightly combined with the surface of CL-20. The results agree well with the theoretical calculations, regardless of the shape or the area distribution of the crystal face. Then, surface elements distribution of CL-20/Al composites was characterized, as shown in Table 2. The distribution of surface elements in 3 locations was measured. Aluminum element content in location 1 is 28.69%, aluminum element content in location 2 is 19.91%, and aluminum element content in location 3 is 13.08%. Therefore, the average value of aluminum element content is 20.56%, which is higher than aluminum element content in the initial sample (15.8). Herein, it is due to the fact that part of the surface of CL-20 is embedded by Al powder, and the blocked CL-20 component cannot be detected.

**Table 2:** Surface element distribution of CL-20/Al composites crystallize in acetate/hexane

Location	C	N	O	Al	Total	
Location 1	20.57	31.92	18.82	28.69	100.00	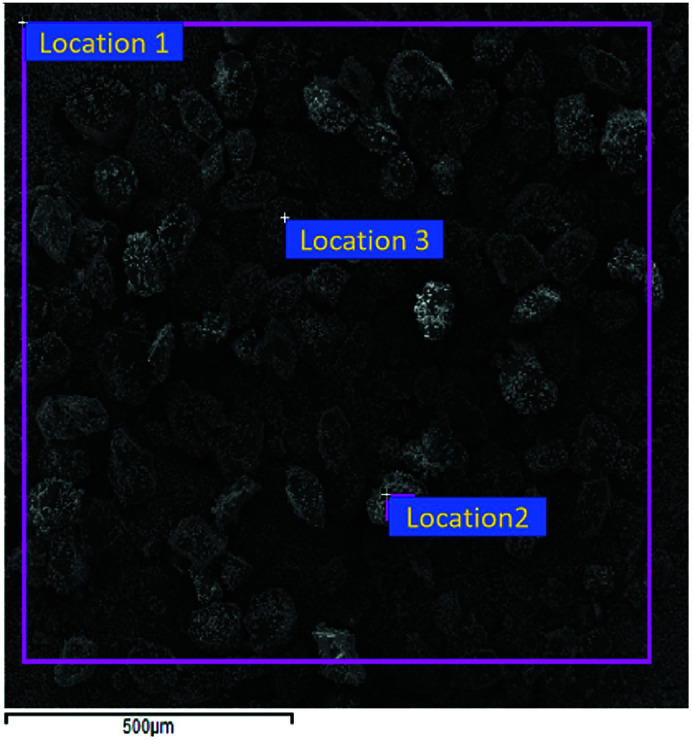
Location 2	14.98	32.96	32.14	19.91	100.00	
Location 3	26.00	36.55	24.37	13.08	100.00	
Average	20.52	33.81	25.11	20.56	100.00	

### 3.4 Interaction mechanism and performance of CL-20/Al composites

To understand the interaction mechanism between CL-20 and aluminum particles in CL-20/Al composites, molecular dynamics calculation was carried out based on the model in Fig. 1, and the radial distribution function (RDF) of O atom in Al_2_O_3_ molecule and H atom in CL-20 molecule in the equilibrium configurations were obtained. The calculation results are shown in Fig. S6.† RDF is the ratio of regional density to average the density in the system. The density of the region near the molecule (*r* value is small) is different from the average density of the system. However, when the molecular distance is far, the regional density should be the same as the average density. In other words, when the *R* value is large, the RDF will be close to 1. *g*(*r*) is usually understood as the geometric distribution of other particles in space (how far away from a given particle) given the coordinates of one particle. Generally, the hydrogen bond length is 1.1–3.1 Å, the strong van der Waals force interaction bond length is 3.1–5.1 Å, and the weak van der Waals force interaction bond length is more than 5.0 Å. As shown in Fig. S6,† there are obvious peaks within the range of 1.1–3.1 Å and 3.1–5 Å for the three systems, and the peak intensity within the range of 2.3–2.6 Å of the three systems is significantly greater than that at 3.1−5 Å, which indicates that there are hydrogen bond and strong van der Waals force interactions between the three crystal faces of Al_2_O_3_ and CL-20. In addition, the hydrogen bond interaction is stronger than the strong van der Waals force.

**Fig. 7:**
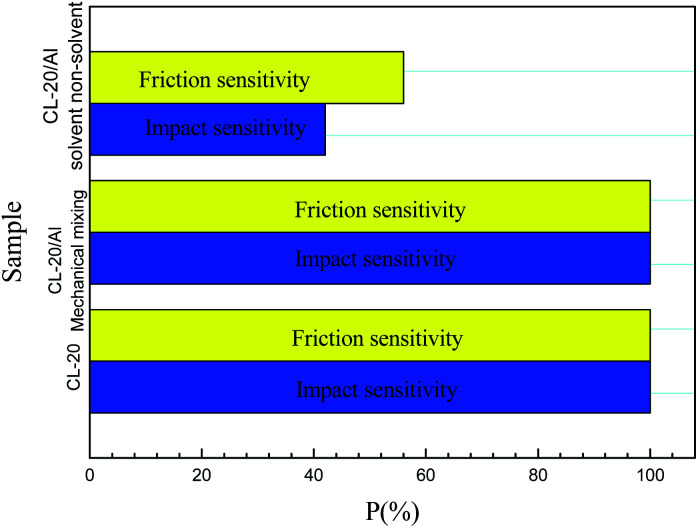
Sensitivities of CL-20 and CL-20/Al composites.

Fig. 7 shows the sensitivities of CL-20 and CL-20/Al composites. From Fig. 7, it can be seen that the impact sensitivity and friction sensitivity of CL-20/Al composites prepared with the mechanical mixing method are 100%. However, the impact sensitivity and friction sensitivity of CL-20/Al composites prepared with the solvent/non-solvent method are 42% and 56%, respectively. Compared with pure CL-20 and CL-20/Al composites prepared through mechanical mixing, the sensitivities decreased significantly. It is thought that the main reason is the change of micro-structure of CL-20 and Al, which leads to the decrease of CL-20 sensitivity. Many aluminum particles are tightly combined with the surface of CL-20 in CL-20/Al composites prepared with the solvent/non-solvent method. Al is a non-explosive material, which can isolate the external friction and impact to a certain extent, as well as protect the internal CL-20. According to Bowden's hot spot theory,^23^ under the external mechanical stimulation, the first material to be stimulated is aluminum powder particles, which will change into heat after the mechanical stimulation applied to aluminum powder. Aluminum powder is an inert material. The heat generated by stimulation will be lost from aluminum powder to CL-20 explosive. This will lead to the reduction of ignition energy of CL-20, and the probability of ignition naturally decrease. Therefore, the sensitivity of CL-20/Al energetic composites with outer cladding and inner embedding structure is significantly lower than that of CL-20 and mechanical mixing composites.

**Fig. 8:**
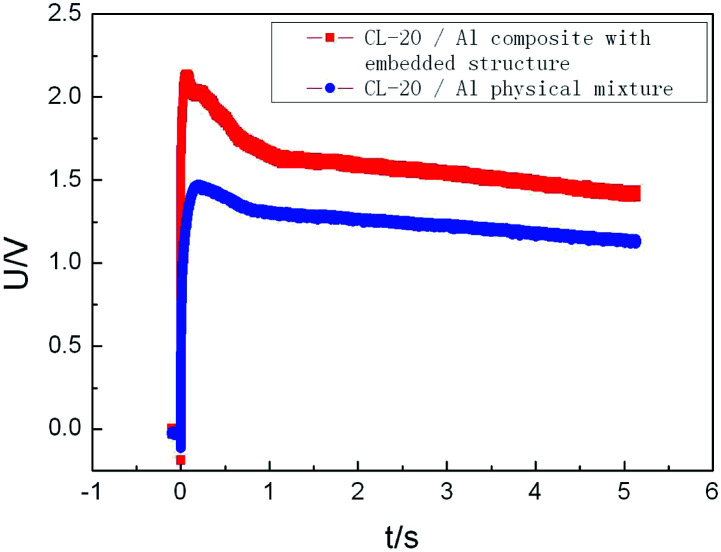
Temperature change of explosive reaction in closed explosion device.

Furthermore, the explosion energy performance of CL-20/Al energetic composites with embedded structure and CL-20/Al physical mixture were compared and studied, and the content of aluminum powder in the composite is 15%. The temperature change of aluminized explosive with CL-20/Al energetic composite and CL-20/Al physical mixture was tested in a closed explosion device, and the test results are shown in Fig. 8. The maximum temperature of CL-20/Al composite based explosive is 2.14 V (791.2 K), and that of CL-20/Al mixture based explosive is 1.47 V (632.5 K). The CL-20/Al composites with embedded structure can improve the reaction completeness of aluminum powder in the composites with aluminum content of 15%, which is an effective way to control the detonation reaction characteristics of aluminized explosives.

## 4 Conclusions

In this paper, the dissipative particle dynamics (DPD) method and the attachment energy (AE) model were applied to predict the mesoscopic and crystal morphologies of CL-20 in solvents. Then CL-20/Al composites were prepared, and the morphology and energy performance were researched. CL-20 and aluminum particles will form the composites of aluminum particles embedded in CL-20 crystal when the content of solvent is zero. In addition, the morphology of CL-20 in CL-20/Al composites is spindle-shaped, and the results agree well with theoretical calculations, and part of aluminum particles are tightly combined with the surface of CL-20. In CL-20/Al composites, there are many aluminum particles embedded in CL-20 crystals. Aluminum particles interact with CL-20 mainly through hydrogen bond and strong van der Waals force. Furthermore the CL-20/Al composites with embedded structure can improve the reaction completeness of aluminum powder and decrease sensitivity.

## Conflicts of interest

There are no conflicts to declare.

## Supplementary Material

RA-010-D0RA07602C-s001
