# Supplementary material for: Multiscale design, interaction mechanism and performance of CL-20/Al energetic composites with embedded structure
Source: RSC Adv. 2020 Dec 18;10(73):44846–53. doi: 10.1039/d0ra07602c (PMC9058612; doi:10.1039/d0ra07602c)
Supplement: RA-010-D0RA07602C-s001 [file RA-010-D0RA07602C-s001.pdf]

Electronic Supplementary Material (ESI) for RSC Advances.
This journal is © The Royal Society of Chemistry 2020

Supplementary information：

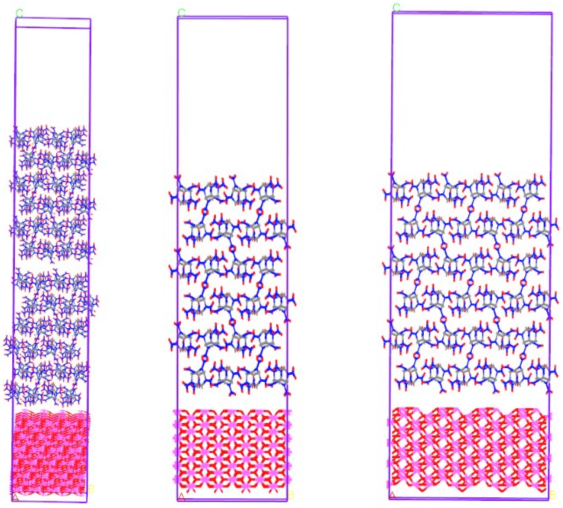

(a) Al_2O_3(001)-CL-20 (b)Al_2O_3(010)-CL-20 (c)Al_2O_3(100)-CL-20

Fig.S1 Initial conformations of Al_2O_3-CL-20

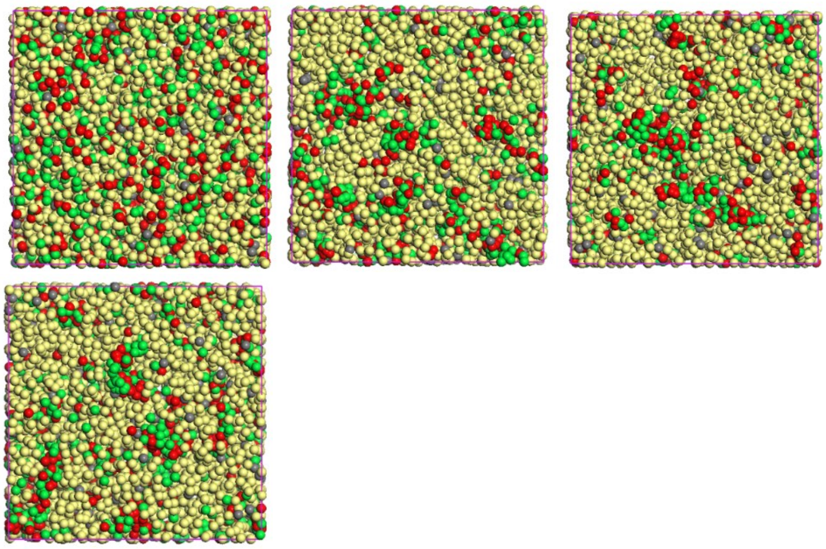

(a) 0 step (b) 80000steps (c) 12000 steps (d) 20000steps

Fig.S2 Mesoscopic morphology of CL-20/Al_2O_3 with 80% solvent volume fraction

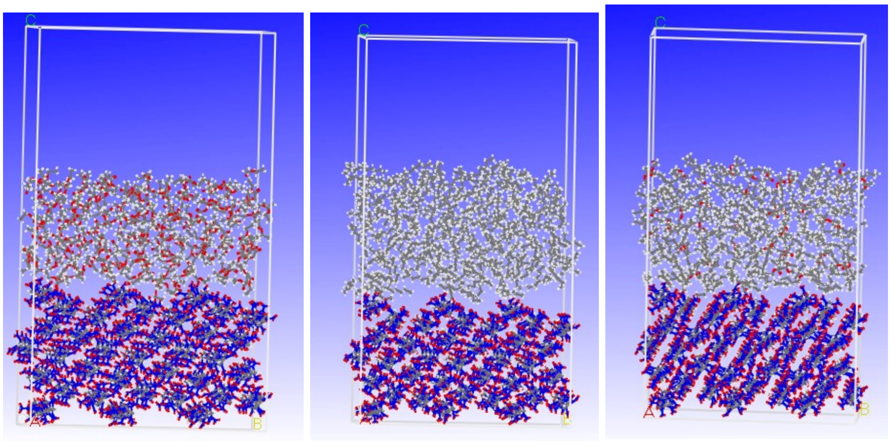

(a) CL-20 (011)- ethyl acetate (b) CL-20 (011)- hexane (c) CL-20 (011)-mixed solvents

Fig.S3 The equilibrium conformations of CL-20 (011) face-solvent

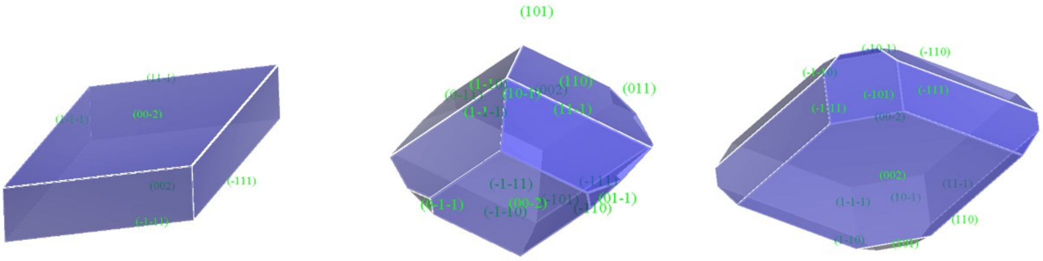

(a) ethyl acetate (b) hexane (c) mixed solvent

Fig.S4 The crystal morphologies of CL-20 crystallization in solvents

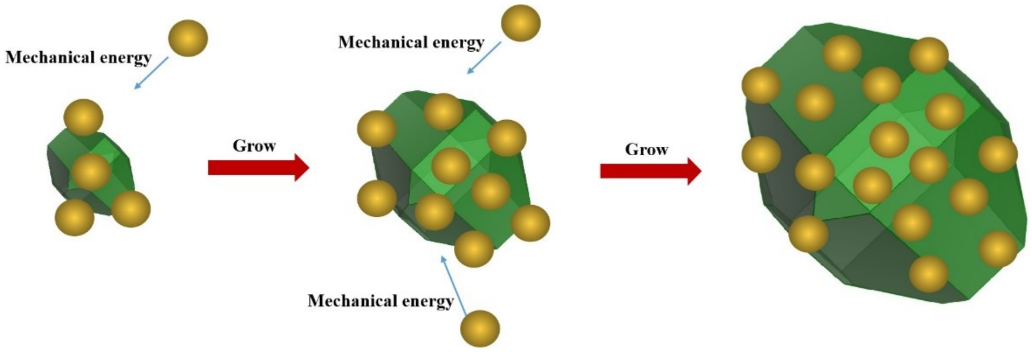

Fig.S5 Morphology prediction of CL-20/Al energetic Composites

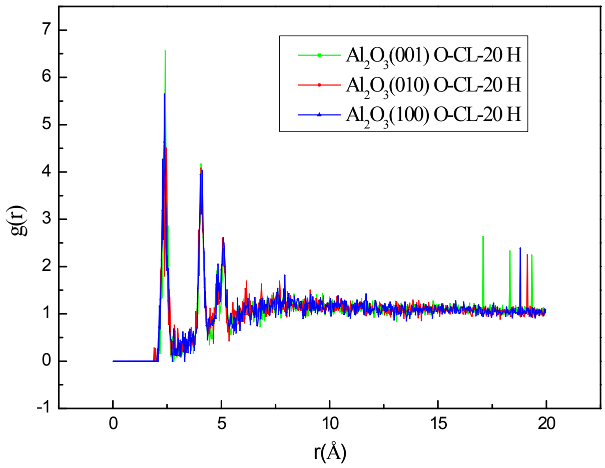

Fig.S6 H—O radial distribution function of CL-20/Al composites

Table S1 Interaction between different parameters of beads

Beads	C	O	Y	Z
C	25	56.53	-49.82	49.03
O	56.53	25	24.48	23.23
Y	-49.82	24.48	25	27.65
Z	49.03	23.23	27.65	25

Table S2 The parameter S values for the crystal habit surfaces of CL-20

(h k l)	(0 1 1)	(10 -1)	(110)	(1 1 -1)	(002)	(101)
A_{acc}	2035.66	2050.22	2081.43	2804.45	1107.82	2650.51
A_{hkl}	1458.87	1551.32	1846.43	1896.65	999.54	2038.27
S	1.39	1.32	1.13	1.48	1.11	1.26

All areas are in Å^2. The solvent-accessible area of an (h k l) face in the unit cell. The surface area of the corresponding (h k l) face.

Table S3 The binding energy between CL-20 crystalline surfaces and solvents

System	Energy types	E/(kJ·mol⁻¹)					
		（0 1 1）	(10 -1)	(110)	(1 1 -1)	(002)	（101）
CL-20/ethyl acetate	E_{total}	-145830.42	-145372.62	-144671.55	-142686.26	-78868.62	-144491.61
	E_{sur}	-132742.00	-132618.44	-132407.02	-130904.06	-66345.46	-132090.34
	E_{solv}	-12064.48	-11622.41	-11288.47	-11278.73	-12061.43	-10977.89
	E_{inter}	-1023.93	-1131.78	-976.07	-503.48	-461.72	-1423.37
Cl-20/hexane	E_{total}	-136893.87	-135361.02	-135561.45	-133483.95	-70096.26	-135085.48
	E_{sur}	-133176.43	-132340.51	-133036.40	-130844.41	-66499.62	-132027.72
	E_{solv}	-2981.80	-2343.77	-1822.31	-2083.98	-2820.00	-2153.16
	E_{inter}	-735.64	-676.74	-702.74	-555.56	-776.64	-904.59
CL-20/mixed solvents	E_{total}	-138490.13	-137264.05	-137429.71	-135876.67	-71302.82	-136980.73
	E_{sur}	-132463.24	-132171.06	-132672.91	-131135.46	-66295.26	-131883.39
	E_{solv}	-4662.58	-4507.88	-3988.72	-4168.88	-4462.65	-4393.47
	E_{inter}	-1364.31	-585.12	-768.08	-572.33	-544.90	-703.87

Note: E_{total} is the single-point energy of the equilibrium structure, E_{sur} is the single-point energy of CL-20, E_{solv} is the single-point energy of acetone in the equilibrium structure, E is the total energy of each structure, vdW is the energy of each structure obtained by the vdW interaction, Eelec is the energy of each structure obtained by electrostatic interaction.

Table S4 Crystal growth paramaters of CL-20 crystal habit faces in solvents

Solvent	(h k l)	E_{int}	A_{acc}	A_{cell}	E_s	E'_{att}	R'_{hkl}
Ethyl acetate	（0 1 1）	-1023.93	2035.66	1458.87	-1428.77	1118.61	6.54
	(10 -1)	-1131.78	2050.22	1551.32	-1495.77	1179.22	6.9
	(110)	-976.07	2081.43	1846.43	-1100.30	768.66	4.5
	(1 1 -1)	-503.48	2804.45	1896.65	-744.46	392.54	2.3
	(002)	-461.72	1107.82	999.54	-511.76	170.96	1
	（101）	-1423.37	2650.51	2038.27	-1850.90	1437.17	8.41
Hexane	（0 1 1）	-735.64	2035.66	1458.87	-1026.48	716.33	1.55
	(10 -1)	-676.74	2050.22	1551.32	-894.39	577.84	1.25
	(110)	-702.74	2081.43	1846.43	-792.19	460.55	1
	(1 1 -1)	-555.56	2804.45	1896.65	-821.45	469.54	1.02
	(002)	-776.64	1107.82	999.54	-860.79	519.99	1.13
	（101）	-904.59	2650.51	2038.27	-1176.29	762.56	1.66
Mixed solvents	（0 1 1）	-1364.31	2035.66	1458.87	-1903.70	1593.54	6.06
	(10 -1)	-585.12	2050.22	1551.32	-773.30	456.75	1.74
	(110)	-768.08	2081.43	1846.43	-865.85	534.20	2.03
	(1 1 -1)	-572.33	2804.45	1896.65	-846.24	494.33	1.88
	(002)	-544.90	1107.82	999.54	-603.93	263.13	1
	（101）	-703.87	2650.51	2038.27	-915.29	501.56	1.92

All energies are in kJ·mol⁻¹, distances are in Å.
